# “Everything…Fell Apart Once COVID-19 Hit”—Leveraging the COVID-19 Response to Strengthen Public Health Activities toward Ending the HIV Epidemic: A Qualitative Study

**DOI:** 10.3390/ijerph192215247

**Published:** 2022-11-18

**Authors:** Samantha A. Devlin, Moctezuma Garcia, Kayo Fujimoto, Camden Hallmark, Marlene McNeese, John Schneider, Moira C. McNulty

**Affiliations:** 1 Department of Medicine, University of Chicago, Chicago, IL 60637, USA; 2 Chicago Center for HIV Elimination, Chicago, IL 60637, USA; 3 School of Social Work, San José State University, San Jose, CA 95112, USA; 4 Department of Health Promotion & Behavioral Sciences, Center for Health Promotion and Prevention Research, University of Texas Health Science Center at Houston, Houston, TX 77030, USA; 5 Division of Disease Prevention and Control, Houston Health Department, Houston, TX 77054, USA

**Keywords:** COVID-19, HIV, EHE, partner services, contact tracing, molecular epidemiology, data systems, public health, information technology infrastructure

## Abstract

COVID-19 caused widespread disruption of activities for Ending the HIV Epidemic (EHE). In this study we assessed public health perspectives on leveraging the COVID-19 response to advance the goals of EHE. We conducted a qualitative study with 33 public health partners in the Midwestern and Southern United States from October 2020 to February 2022. Participants were asked how the strategies developed for COVID-19 could be applied to the HIV epidemic. Interviews were recorded, transcribed, and examined using rapid qualitative analysis. Four themes emerged: (1) Rebuilding teams and adapting culture for success in EHE activities; (2) Recognizing and modernizing the role of disease intervention specialists (DIS); (3) Enhanced community awareness of the public health role in disease response and prevention; and (4) Leveraging COVID-19 data systems and infrastructure for EHE activities. The COVID-19 pandemic called attention to the dearth of public health funding and outdated information technology (IT) infrastructure used for HIV activities. It also led to greater public health knowledge, including increased familiarity with partner services and molecular epidemiology of HIV, and opportunities to develop new data systems for surveillance that can be applied to efforts for EHE.

## 1. Introduction

Early in the COVID-19 pandemic, many studies focused on how lessons learned from the decades of trying to end HIV could be applied to both the public health response and individual behavior for reducing COVID-19 transmission [1,2,3,4,5,6,7,8]. As the pandemic progressed, focus shifted to how clinical and community-based HIV services were disrupted [9,10,11,12,13,14]; different strategies were developed to overcome challenges such as at-home or video-based HIV testing technology [15,16] and delivery of care via telemedicine [17,18,19,20,21,22]. Although existing systems related to HIV enhanced efforts to respond to the public health emergency, the pandemic also revealed deficits in our public health and healthcare infrastructure, necessitating a re-evaluation of the methods used to understand HIV care quality and inform programming [23]. Overall, COVID-19 exposed the need to strengthen HIV/STI surveillance, programs, and services [24]. 

Ending the HIV Epidemic (EHE) in the United States (U.S.) is a national plan to reduce the number of new HIV infections by 90% by the year 2030 [25]. The plan focuses on high priority jurisdictions, calling for cross-agency coordination to leverage all existing tools and resources for the HIV response, with four core pillars: Diagnose, Treat, Prevent, and Respond. In addition, many U.S. jurisdictions have local initiatives to end HIV transmission [26]. The COVID-19 pandemic disrupted the HIV response, yet forced the public health system to quickly adapt tools for surveillance, contact tracing, public health messaging, and eventually vaccine delivery. The strategies developed in response to COVID-19 can be leveraged to improve HIV surveillance and service delivery, particularly for achieving the goals of the EHE initiative. In this qualitative study, we aimed to understand public health practitioners’ and partners’ views on how the strategies and systems that were developed for the COVID-19 response could inform or be adapted for the HIV epidemic response within the Midwestern and Southern U.S. 

## 2. Materials and Methods

(1) Study Design

We conducted an exploratory qualitative study with public health partners in the South and Midwest to investigate the potential ways the COVID-19 response can be legeraged for the goals of EHE. We orginally developd a semi-structured interview guide to understand gaps in the monitoring and evaluation systems for EHE. With the onset of COVID-19, we added a section to the interview guide to gather information about the impact of the COVID-19 pandemic on ongoing and planned HIV-related activities and implications for future EHE work (see Supplementary Material). Participants were asked about the impact of becoming part of the essential public health workforce to enhance COVID-19 response efforts in their local community. Participants were asked how the COVID-19 response can be leveraged for the goals of EHE, particularly in terms of data systems or other lessons learned that can inform or be adapted for the HIV epidemic response.

(2) Recruitment

Purposive sampling was used in collaboration with contacts at local and state public health departments and planning committee members of city-, county- and state-level HIV elimination initiatives to identify public health partners (e.g., health department, community-based organization, and planning committee or council members) in the South and Midwest. Sites were chosen based on EHE priority jurisdictions with high HIV prevalence and preexisting research partnerships. Participants were eligible if they were currently or formerly directly involved in HIV elimination activities (i.e., strategic planning, service delivery, surveillance, etc.) as an employee, council, or committee member within the relevant geographic areas, were aged 18 years or older, and able to speak and understand English. Potential participants received an initial recruitment email that explained the nature of the study. Recruitment occurred over a two-year period to include as many participants as possible. We aimed to recruit at least 30 participants and a maximum of 50 participants, with near-equal representation from the geographic locales and wide representation in types of roles participants held in relation to HIV elimination initiatives. The final sample size was influenced by the qualitative analysis, with interviews concluded when theme saturation was reached (see sections on Data Analysis and Quality Considerations below).

(3) Data Collection

Confidential interviews with interested participants were conducted by video conference or telephone (45–60 min). Participants interviewed through video conferencing were informed that only audio was being recorded and had the option to turn off their webcam. Due to remote interviewing processes, it was sometimes difficult to assess nonverbal cues expressed by participants. However, our study was conducted among people who were familiar with technology, so we did not encounter any logisitical barriers among participants. Indeed, the use of remote interviewing methods was appropriate in the setting of the ongoing COVID-19 pandemic, which limited in-person contact. Additionally, remote interviewing procedures resulted in greater flexibility and ability to include particpants with busy work schedules. After completing the interview, participants were asked to complete a brief demographic survey.

(4) Data Analysis

Interviews were audio recorded and transcribed verbatim. We conducted a rapid qualitative analysis (RQA) [27], which has been shown to produce valid findings consistent with traditional in-depth thematic analysis [28,29]. RQA involves summarizing interviews and creating matrices based on the summaries to identify key points and potential themes and to ensure quality and consistency of data collection; it reduces time spent on analysis and can improve both the efficiency and accuracy of data analysis [30]. Three researchers summarized each interview transcript using a structured template. Transcript summaries were assessed for commonalities related to how COVID-19 has impacted current HIV-related activities and how lessons learned could be applied for the goals of EHE. The summary template was iteratively examined and revised as needed to reflect the major domains found across interviews. All transcript summaries were reviewed by at least two researchers who discussed discrepancies to agreement. A researcher then consolidated the summaries into matrices and found key themes based on clustering of domain groupings. We selected representative quotes to illustrate key themes.

(5) Quality Considerations

Saturation of themes was determined when there was a high prevalence of domain groupings within the matrices and no emergence of new themes. Each theme was distinct and had enough supporting data to be considered relevant to our findings. The study was conducted by a team with qualitative research expertise who received training in qualitative methods and used standardized approaches for data collection and analysis, lending credibility to the study. Transcripts were assessed for accuracy and corrected based on audio files when needed. Additionally, we presented preliminary findings to a subset of study participants, who provided invaluable feedback and ultimately approved of our conclusions.

(6) Ethical Considerations

All participants provided verbal informed consent to participate in the study and were offered a $100 USD gift card upon completion of the interview. Participant identification numbers were assigned for each participant to protect their identity and ensure anonymity. This study was approved by the Institutional Review Board at the University of Chicago.

## 3. Results

Between October 2020 and February 2022, a total of 33 interviews (19 based in the South and 14 based in the Midwest) were conducted with participants involved in EHE efforts. Most participants were non-Hispanic White (72.0%), cisgender female (60.0%), age 40 or older (60.0%), and worked at a public health department (84.0%) (Table 1). Participants included employees of varying job roles and experience levels, including current and former directors and chiefs of departments/bureaus, program managers, epidemiologists, public health advisors, and data analysts.

### 3.1. Themes

Four major themes emerged across interviews regarding how the lessons of COVID-19 can be leveraged for the goals of EHE: (1) Rebuilding teams and adapting culture for success in EHE activities; (2) Recognizing and modernizing the role of disease intervention specialists (DIS); (3) Enhanced community awareness of the public health role in disease response and prevention; and (4) Leveraging COVID-19 data systems and infrastructure for EHE activities. 

#### 3.1.1. Rebuilding Teams and Adapting Culture for Success in EHE Activities

Many participants commented on how COVID-19 either “*put everything on hold*” (ID 1) regarding HIV activities or how “*everything kind of fell apart once COVID-19 hit as far as our EHE responses*” (ID 4). Participant ID 8 indicated: “*I feel like we’ve really slid backwards, probably, a couple years. COVID has really just blown apart anything we were working on for HIV.*” 

HIV surveillance and evaluation teams were “*stretched really thin*” and “*that makes getting things done extremely difficult and burnout extremely high. Additionally, in addition to the vacancy rate, all the remaining functioning staff have COVID assignments on top of their HIV assignments*” (ID 20). Despite the staffing challenges, some participants viewed the pandemic as an opportunity to rebuild their teams with PrEP and HIV linkage to care strategies in mind.

*We’ve had a lot of vacancies in our program over the past year which have been delayed even further because of COVID. We don’t have a very large team right now. But, as we are rebuilding this team, we are also thinking and strategizing about how we can better incorporate these strategies of PrEP* [pre-exposure prophylaxis] *and HIV Linkage to Care within our partner services work*.

(ID 23)

Due to the reassignment of staff on various teams for the COVID-19 response, participants recognized the value of inter-team collaboration, which would benefit HIV activities as well, as this participant elaborated:

*COVID really forced us out of that* [working in silos within the health department] *and made us more aware of how content-driven our staffing structures are, and how it really doesn’t work very well when you are trying to like mobilize for an emergency, you really need all types of people with different skills and backgrounds*… [we were] *suddenly able to work really directly with medical directors and epidemiologists and people that* [we’ve] *always worked with before, but in a very limited capacity*… [COVID-19] *shifted us to kind of day-to-day partnerships and using data and information and all of this stuff like much more effectively because we had people at the table who were very close to that piece of the* [health department] *machine*.

(ID 24)

The same participant also noted how COVID-19 impacted the culture at health departments and highlighted the need to prioritize activities to end HIV rather than fulfilling administrative duties.

*I think a… lesson learned* [from COVID-19] *is… trying to move away from that culture of grant management. I think there’s a time period where that was really, really important to…be able to perform a certain way and to be able to show that you’re checking all the boxes, following all the rules. And at the end of the day, that’s not what ends an epidemic*.

(ID 24)

#### 3.1.2. Recognizing and Modernizing the Role of DIS

COVID-19 resulted in recognition of the pivotal role that DIS, who perform contact tracing, play in directly engaging with the community and controlling communicable diseases. Nearly all participants explained how DIS and others working on HIV activities were reassigned to the COVID-19 response due to their relevant experience.

*The COVID pandemic has impacted our program quite substantially. For many jurisdictions, HIV partner services aligns very closely with the work that’s needed for COVID contact elicitation and notification, so many staff are pulled from their HIV/STI programs to assist in the COVID work…it has left quite a big gap in our program work*.

(ID 23)

However, DIS continued their important work, even in the midst of the COVID-19 pandemic.

*There was a period of time where we talked a lot about the heroes of COVID. And the frontline health care workers. And I think the people who were definitely not in the spotlight were HIV prevention outreach workers…long before the vaccines were available, were going out in the community still, delivering services, wearing masks, and using at the time our best safety precautions. But those providers have sustained services throughout the pandemic.*

(ID 33)

DIS adapted their techniques and utilized COVID-19 as an opportunity to find alternative ways for community outreach when traditional in-person contact tracing was not an option. DIS primarily used web-based services to engage with clients, including “*strategies that we have now because of COVID. We are utilizing…telephone interviews, video, apps…so I think that that works really well and I wanted to continue that process* [for HIV].” (ID 28)

*There has been a much greater emphasis* [during the COVID-19 pandemic] *on using web-based services, social…networks of Facebook, and mainstream social networks, but also hook up apps and those types of resources. People have been more restricted in terms of how much community outreach they can do…if people aren’t gathering in public en masse in the way that they used to, then you got to find other ways to engage and connect with them…That’s been probably one of the biggest shifts is less in-person outreach and education and more finding ways to connect with people electronically* [for HIV activities]…*There’s a lot of money right now going into recruiting and engaging people into testing services by using social media, by being very targeted and intentional in that way.*”

(ID 33)

Additionally, participants hoped the public health funding resulting from COVID-19 that supported contact tracing would allow DIS to expand partner services beyond traditional expectations to meet the goals outlined in the EHE plan during the post-pandemic era.

*We hope that with*… [COVID-19]…*there* [will be] *new investments in disease intervention* [for]…*more than just eliciting partners and getting the partners taken care of but also linking people to other services that they might need, maybe do some health equity* [work]…*There is optimism that it could be evolved and not solely focused on how many partners were identified, how many partners were followed up on…I think there is optimism that we will evolve partner services to have more impact than it’s having pre-COVID*.

(ID 25)

#### 3.1.3. Enhanced Community Awareness of the Public Health Role in Disease Response and Prevention

COVID-19 called community attention to the role of the public health department in leading the way to greater awareness and understanding at both the individual and community level.

*We* [public health department] *were* [in] *a leadership role as perfecting a unified message, which I think became very clear in COVID. Like, you know, a kind of a clear-headed, unified, this is a public health message. And, I think we got visibility there. I think, prior to that, we were kind of invisible, people knew* [disease intervention specialists]*… because we were the awful people who ran after them for* [HIV/STI services]. *We were doing mobile vaccinations, we were doing mobile testing, reaching out* [to] *providers, and a lot more calls. And I think* [that resulted in] *recognition from the public that public health is pretty important* [for] *the community.*

(ID 21)

Many participants commented on how COVID-19 also led to increased consciousness regarding public health and disease management within the general population. Several participants reported that clients now understand the significance of HIV disease intervention and partner services because of the similarities with contact tracing used as part of the COVID-19 response.

*We’re in a really interesting time for HIV prevention and public health. Because the general knowledge about disease intervention, the consciousness of it is so much higher now because of COVID. Because when you describe HIV prevention to people, they will say…“That sounds a lot like what we do with COVID-19.”*

(ID 33)

There is also a better understanding of molecular epidemiology and cluster detection due to the pandemic. Increased awareness may particularly benefit the ‘Respond’ pillar of EHE, which emphasizes approaches to detect rapid HIV transmission and promptly respond. Participant ID 27 indicated that the public health department’s response to COVID-19 and awareness of variants enhanced the community’s understanding of molecular epidemiological data and how it applies to molecular HIV cluster detection:

*People start talking about different serotypes, or Delta variant, or Omicron variants. So, people start understanding what the sequences is, genetic sequences. I think even in this aspect COVID was instrumental. I do believe that understanding of overall use of or building the internal knowledge*… [of] *molecular epidemiology will increase across the board, across all of the jurisdictions. This is…one of the strategies, right, under the EHE is…to do a molecular cluster analysis.*

Participants noted that the challenge would be taking advantage of the momentum and heightened community awareness of COVID-related public health activities to develop effective public health messaging for HIV. The elevated attention given to the COVID-19 pandemic must be mirrored for EHE activities to attain success. The following participant further elaborated:

*I think the challenge for HIV and taking the lessons that maybe we have as a society from COVID and apply that to HIV is, people don’t always think of HIV as an ongoing epidemic, or a major health issue anymore…That’s a testament to the effectiveness of HIV prevention services and…to the value of antiretroviral therapies that people don’t associate…HIV* [with]…*a death sentence anymore. Those messages have been effective and gotten out there. And so… once we feel like COVID is more under control, how do we then take those lessons and then apply them to the other major diseases that we’re trying to address?*

(ID 33)

Although there was consensus on how COVID-19 exposed the importance of investment in the field of public health, one participant thought that benefit might have been somewhat negated by the politicization of public health messaging throughout the COVID-19 pandemic.

*The only thing, I think that has come out of it* [COVID-19] *is I think people have gotten attention to the field of public health and the need to invest in it. However, I think that benefit, unfortunately, might have been offset by the mistrust and confusion and political negatives around science that has come up.*

(ID 8)

#### 3.1.4. Leveraging COVID-19 Data Systems and Infrastructure for EHE Activities

Participants emphasized how data “*drives almost all of the decisions we make about the work that we do, the kinds of programs we fund, the priorities we set for populations of greatest need and geographic areas of greatest need*” (ID 26). Yet, many participants reported that COVID-19 revealed where health departments “*were lacking*” with systems that were “*not designed to deal with this large amount of data*” and what would usually take “*15 min, now takes two hours to run.*” COVID-19 demonstrated that existing data systems were outdated and hindered the ability to respond quickly to highly communicable diseases. 

Participants emphasized how COVID-19 “*revealed a lot of lack attention in the area of our IT infrastructure*” (ID 3) and forced them to “*reexamine why things are built the way they are and why we function the way we do*” (ID 24). COVID-19 highlighted a dire need for funding to enhance outdated infrastructure because “*the technology just in general needs to be updated and improved for disease reporting and everything else*” (ID 6). Participant ID 4 provided greater insight into the current infrastructure for data management:

*COVID has really highlighted how…our infrastructure on electronic lab reporting is not great. For example…a lot of people report to us still via a fax machine, which just is a lot of extra work for manual data entry…for surveillance purposes. So, but one of the benefits of COVID is that we’re really trying to address all of those issues, to really address the timeliness of getting data for COVID response ‘cause it’s a really short window that we have to respond. But the side benefit of that is it’s gonna improve our infrastructure for all of our reportable conditions*.

Overall, participants viewed the pandemic as an opportunity to build capacity and infrastructure for EHE activities. While finding “*ways we can adopt data systems that we saw worked really well during COVID for HIV… [is] kind of an ongoing, never-ending question*” (ID 24), participants felt “*it should look more like…grants did for COVID, where they gave a huge bolus of dollars locally…into health departments to increase their data infrastructure…we’ve never seen anything like that for HIV*.” (ID 5) Participant ID 5 further indicated:

*We’re trying to use COVID as an opportunity to take…the resources that are certainly prioritized for COVID and have it…undergird our data capacity in a way that, yes, we can have more responsiveness toward COVID. But it could also serve as creating more capacity and efficiency for all of our other reportable conditions like HIV. So, I think we’re–even though we don’t have current resources, what we are doing as a department is also trying to leverage this current COVID opportunity, lots of resources going to COVID right now, they may not last forever, but if there’s a way to help with some of our core infrastructure building, then we’re trying to do that.*

One of the biggest areas of improvement and opportunity was in data systems used for contact tracing:

*Because of COVID, I think we’ve had to touch some different systems that work more efficiently than the things that we have in HIV. So one of the things I did before was work on COVID contact tracing and we used a system…which was really fantastic compared to some of the stuff that I’ve ever seen in public health before.*

(ID 24)

COVID-19 also demonstrated the need to “*monitor things actively and routinely…now that we have the capabilities and the knowledge in creating dashboards, we’re trying to transition to that [for HIV activities]*” (ID 1). Some data systems developed during the pandemic can be utilized for HIV with external partners as well.

*Some of the systems that we had in place for COVID* [were] *just very user-friendly, very intuitive…I think there*… [were] *some really good innovations to develop systems that partners could use that would integrate with the health department that would be user-friendly and compatible with other systems* [for HIV activities].

(ID 25)

In particular, participants mentioned how “*the reporting of COVID to follow up with the clients, that timeframe was very short and if we were able to leverage the lessons learned there to HIV and STI intervention, we would be able to shorten the timeframe from data reporting to client follow up*” (ID 23). Another participant stated that the “*electronic feed process [used] for COVID… [would] be really cool for HIV*” (ID 14) because of the reduced need for manual data entry and closer to real-time reporting resulting in part from “*adoption of the CSV to HL7 tool [for reporting COVID-19 tests] that has tremendously helped us get these labs into our system faster for intervention.*” (ID 1) Participant ID 5 commented:

*That used to be, you know, days, maybe even weeks in some worse case scenarios* [to perform outreach to someone newly testing positive for HIV]. *For COVID, we’ve been able to cut that down tremendously, and that’s because we were able to build out an electronic way to be able to do that* [notify the contact tracing team in as close to real time as possible]. *So that’s what I mean as one example of my hope for what could happen with additional resources for HIV.*

## 4. Discussion

To our knowledge, this is the first qualitative study to interview public health practitioners and partners to explore how the lessons learned from the COVID-19 pandemic can inform or be adapted for the HIV epidemic response. Participants from the Midwestern and Southern U.S. commented on how COVID-19 disrupted HIV work and exposed a lack of infrastructure for public health activities, yet simultaneously highlighted the role and important work of DIS for contact tracing, as well as created opportunities for increasing public health knowledge and applying lessons learned from the pandemic to ongoing and future HIV efforts. 

The reassignment of DIS and other HIV staff has been described in other studies as well [31]. Despite the increased vacancies and setbacks, participants viewed the pandemic as an opportunity to better incorporate HIV intervention strategies when rebuilding their teams and DIS were able to demonstrate the invaluable role they play in terms of direct community engagement and outreach. In particular, participants noted how clients have a better understanding and appreciation of the role of contact tracing. Similarly, participants reported that different variants of COVID-19 resulted in more familiarity with HIV molecular epidemiology among clients. With this increased awareness, it is possible that community members may feel more comfortable cooperating with partner services and being receptive to cluster detection and response efforts, which is a key component of the ‘Respond’ pillar of EHE.

In addition to disrupting HIV activities, the pandemic revealed a longstanding lack of funding and attention devoted to the field of public health, including outdated IT infrastructure and the need for enhanced database systems and processes for HIV prevention and care activities, which hampered community coordinated responses when they were needed most in times of crisis to serve marginalized and underserved populations and communities [32,33]. Data were described as crucial for equitable service delivery, which is true for both the COVID-19 and HIV responses. Resources available during the pandemic led to the development of innovative and efficient surveillance and contact tracing database systems for COVID-19. The systems developed for COVID-19 are described by our participants as a distinct advancement over those for HIV-related activities, particularly in method and speed of data reporting. Although our participants described the immense potential of new and innovative systems, they also indicated that these advancements have not yet fully translated into widespread improvements for EHE activities. Ensuring that is the case will require additional funding and investment in public health informatics systems.

Other lessons that COVID-19 imparted that can be applied to EHE activities included creative solutions to follow up with clients during times of social distancing. The utilization of mobile and social media platforms has been shown to be effective for HIV intervention strategies, including support for treatment adherence throughout the pandemic [34]. These methods for contacting clients and eliciting partners will likely continue for the foreseeable future, as patients may be more comfortable discussing sensitive information in the privacy of their homes. Indeed, other studies have echoed how COVID-19 accelerated public health funding and led to new HIV care paradigms, including the use of telehealth and improved accessibility of Ryan White HIV/AIDS Program services [35]. Additionally, the lessons learned from the COVID-19 response can potentially address issues of equity, justice, and access to care within the public health system for people living with HIV [36,37,38]. 

This study has several limitations to acknowledge. The sample size was relatively small for transferable findings but suitable for a qualitative study. Despite prolonging the recruitment period, there were still partners who could not participate due to COVID-19 responsibilities. Participants from the South and Midwest were included, but we did not compare regional differences of the pandemic’s effects. It is also possible that the specific time a participant was interviewed could have impacted their responses (e.g., during a wave of COVID-19 infection within their community). However, we were able to find consensus among participants and reach theme saturation. Future studies should explore how the lessons learned from COVID-19 could be applied for EHE activities in other high HIV burden areas throughout the U.S. to further assess any regional-specific considerations.

## 5. Conclusions

The COVID-19 pandemic disrupted ongoing HIV-related activities at public health departments, draining resources and presenting staffing challenges. However, the pandemic also called attention to the dearth of public health investment in information technology and public health infrastructure used for HIV prevention and care activities. The often-overlooked work of DIS was also acknowledged and better understood, which will likely have a positive effect on future EHE efforts. In response to COVID-19, database systems were developed for monitoring and evaluation that can also be applied to HIV. These strategies included web-based services for client follow-up and improvements to electronic laboratory reporting systems or processes. Because of the pandemic, public health practitioners and partners can take lessons learned from the COVID-19 response and apply them to HIV work, including leveraging the innovative strategies, updated systems, and increased public health knowledge for the goals of EHE.

## Figures and Tables

**Table 1:** Participant demographics (*n* = 25 ^1^).

Category	*n* (%)
**Race** ^2^	
Black/African American	4 (16.0)
White	18 (72.0)
Asian/Asian American/Pacific Islander	3 (12.0)
Native American/American Indian/Alaska Native/Indigenous	2 (8.0)
Other	1 (4.0)
**Hispanic/Latino Ethnicity**	
Yes	6 (24.0)
No	19 (76.0)
**Age**	
18–29	1 (4.0)
30–39	9 (36.0)
40–49	7 (28.0)
50–59	6 (24.0)
60 or older	2 (8.0)
**Gender**	
Cisgender female	15 (60.0)
Cisgender male	9 (36.0)
Transgender male	1 (4.0)
**Organization**	
Public health department	21 (84.0)
Non-profit organization	1 (4.0)
Other government agency or planning committee	3 (12.0)

^1^ 25/33 participants completed the demographic survey. ^2^ participants could select more than one race.

## Data Availability

The data presented in this study are unavailable for confidentiality reasons, as the data contain potentially identifiable information.

## Supplementary Materials

The following supporting information can be downloaded at: https://www.mdpi.com/article/10.3390/ijerph192215247/s1, Table S1: Subset of Relevant COVID-19 Questions asked of Qualitative Interview Participants.
