# Supplementary material for: “Everything…Fell Apart Once COVID-19 Hit”—Leveraging the COVID-19 Response to Strengthen Public Health Activities toward Ending the HIV Epidemic: A Qualitative Study
Source: Int J Environ Res Public Health. 2022 Nov 18;19(22):15247. doi: 10.3390/ijerph192215247 (PMC9690919; doi:10.3390/ijerph192215247)
Supplement: Supplementary file 1 [file ijerph-19-15247-s001.zip › ijerph-1986651-supplementary.pdf]

Table S1. Subset of Relevant COVID-19 Questions asked of Qualitative Interview Participants

Introductory Text	We realize this is a difficult time with many changes taking place related to COVID-19. Feel free to tell us throughout the conversation how COVID-19 has impacted you and your organization in relation to EHE.
Questions	What are some of the challenges you face in your role?<br>• Has the COVID-19 pandemic presented new challenges?
	Can you tell me what data sources your organization currently uses to monitor the HIV epidemic?<br>• Do you feel that your organization has sufficient resources to optimally use the data?<br>• What are some of the facilitators?<br>• What are some of the barriers?
	Describe current collaborations or partnerships between your organization and others around sharing of data.
	Are there ways you think the COVID-19 response can be leveraged for the goals of EHE, in terms of data systems or other lessons learned that can inform or be adapted for the HIV epidemic response?

Abbreviations: EHE: Ending the HIV Epidemic
